# The effects of different intensities of aerobic exercise for 8 weeks on neurogenesis, depression, and anxiety in young mice

**DOI:** 10.7586/jkbns.25.006

**Published:** 2025-05-08

**Authors:** Mi Yang Jeon, Quan Feng Liu, Chi Yang Yoon, Bong Gyu Kim, Ji Hyun Kim, Ha Jin Jeong, Songhee Jeon

**Affiliations:** 1 College of Nursing, Sustainable Health Research Institute, Gyeongsang National University, Jinju, Korea; 2 Department of Neuropsychiatry, Graduate School of Oriental, Dongguk University, Ilsan, Korea; 3 College of Nursing, Gyengsang National University, Jinju, Korea; 4 Department of Nursing, Asan Medical Center, Seoul, Korea; 5 Department of Nursing, Gwangju Health University, Gwangju, Korea; 6 Department of Biomedical Sciences, Center for Glocal Future Biomedical Scientists at Chonnam National University, Gwangju, Korea

**Keywords:** Exercise, Mice, Neurogenesis, Depression, Anxiety

## Abstract

**Purpose:**

This study aimed to assess the impact of aerobic exercise at different intensities over an eight-week period on the expression and activation of cortical synaptic proteins, with the potential to reduce anxiety and improve memory in young mice.

**Methods:**

Seven-week-old C57BL/6 mice were subjected to treadmill exercises at low (n = 10), moderate (n = 10), and high intensity (n = 10) for eight weeks. Behavioral assessments were conducted to evaluate anxiety and cognitive function. To explore the underlying mechanisms, we measured the phosphorylated levels of extracellular signal-regulated kinase (ERK), cyclic adenosine monophosphate response-binding protein (CREB), protein kinase (AKT), adenosine monophosphate activated protein kinase (AMPK), synapsin (S9, S549, S609), and PSD-95 in the cortex, as these are associated with synaptic strength. Additionally, the expression of doublecortin (DCX), a neurogenic factor, was analyzed in the hippocampus.

**Results:**

Exercise led to reductions in depressive and anxiety-related behaviors and elevated the levels of phosphorylated ERK, CREB, AKT, AMPK, synapsin (S9, S549, S609), and PSD-95 in the cortex of young mice. Furthermore, exercise increased DCX expression in the hippocampus. Moderate-intensity exercise yielded more pronounced effects than other intensities.

**Conclusion:**

The findings of this research indicate that consistent moderate-intensity exercise increases synaptic strength and reduces depression and anxiety in young mice by activating multiple factors.

## INTRODUCTION

The outbreak of the coronavirus disease 2019 (COVID-19) pandemic has seriously affected the quality of human life. In particular, previous studies show that the pandemic, along with the resulting lockdowns, school closures, and social distancing measures, had a significant impact on adolescents' mental health [1]. The pandemic may negatively impact adolescents’ mental health through stress transferred from family members, changes in context and policy, and the disruption of daily routines [2,3].

Adolescents who had above-average mental health prior to the pandemic have reported increases in emotional issues, conduct disorders, hyperactivity, and peer relationship problems, along with a decrease in prosocial behaviors during this period [4]. Numerous studies affirm that physical exercise provides a wide range of beneficial physiological, psychological, and neurocognitive effects. These benefits include reductions in stress [5], anxiety [6], and depression [7], as well as positive effects on cognitive functions such as enhanced executive and memory abilities [8]. Exercise can function as a cognitive enhancer by boosting neuroplasticity, neurotrophic signaling, and monoaminergic transmission, leading to improved cognitive performance both in the short and long term [8].

Many studies have shown that exercise-induced improvements in cognitive function (CF) have been associated with local and systemic expression of growth factors in the hippocampus. van Praag et al. [9] found the survival of newborn cells in the adult mouse dentate gyrus (DG), a hippocampal region important for spatial recognition, is enhanced by voluntary wheel running. Similarly, spatial pattern separation and neurogenesis in the DG are strongly correlated in three-month-old mice following 10 weeks of voluntary wheel running [10], and the development of new neurons in the DG is coupled with the formation of new blood vessels [11].

In the adult mammalian hippocampus, new neurons continue to be generated in the subgranular zone of the dentate gyrus (DG). Rodent studies suggested that exercise has the potential to remodel dendritic arbors. Exercise increased dendritic length and the mean number of branches on neurons in the DG [12]. Additionally, exercise increased spine density and length, as well as synaptic markers in the medial prefrontal cortex (mPFC) [13].

In studies conducted on rodents, exercise has been reported to affect brain function by increasing neurotrophic factors and enhancing learning and memory. Nonetheless, the effect of exercise on dendritic plasticity may be stronger in adolescents compared to adults. In the study by Eddy & Green [14], two weeks of running wheel exercise resulted in a higher density of dendritic spines and shorter dendritic branch lengths in the adolescent exercise group compared to the non-exercise adolescent group. No differences were observed between the adult exercise and non-exercise groups.

Vysniauske et al. [15] reported that extended durations of exercise were associated with larger effect sizes, whereas exercise intensity did not exhibit any moderating influence. However, Den Heijer et al. [16] suggest that 30 min of individually adapted daily exercise represents an appropriate duration and frequency. Grassman et al. [17] proposed that 30 min of exercise can enhance CF. This notion is further supported by Suarez-Manzano et al. [18], who recommended engaging in 20-30 min of moderate-intensity exercise (40%-75%) for immediate benefits, and participating in sessions of over 40% intensity at least three times a week for more than five weeks to achieve long-term cognitive improvements. Halperin et al. [19] also support the finding of long-term enhancements in CF metrics. However, the recommendations concerning exercise intensity and duration are based on existing literature, which currently lacks sufficient evidence to fully validate these suggestions. To our knowledge, the effects of exercise intensity on children's brain structure, neurogenesis, and anxiety and depression have not been clearly established. Therefore, this study attempted to determine the effects of exercise intensity on brain structure, neurogenesis, anxiety, and depression in young mice.

## METHODS

### 1. Study design

A pretest-posttest, equivalent control group, experimental design was used in this study.

### 2. Experimental animals

Seven-week young male C57BL/6 mice were purchased from OrientBio (Seongnam, Korea). The animals were kept at a stable temperature of 21 ± 1°C and a relative humidity of 55 ± 5%, with a 12-hour light and 12-hour dark cycle. To examine nutritional status, weight gain and food intake were recorded every week at the same time for eight weeks.

### 3. Measurements

#### 1) Behavior tests

Open field test (OFT) was performed inside a box located in a dark room, measuring 40 cm x 40 cm x 40 cm. This box was segmented into 16 equal squares, referred to as “zones.” During each trial, a single mouse was placed at the center of the arena, and both the number of crossings into the central zone and the total distance traveled were recorded over a duration of 10 min. Following the testing of each mouse, the box was meticulously cleaned with 70% alcohol. The researcher conducting the experiment remained unaware of the group assignments. Data regarding the total distance traveled and the time spent in each zone were automatically analyzed using the ANY-maze video tracking system (Stoelting Co., Wood Dale, IL, USA).

#### 2) Elevated plus maze (EPM) test

EPM test features four arms, each measuring 35 cm by 6 cm, arranged in a plus shape and elevated 50 cm above the ground. Two opposite arms are enclosed with 15 cm high walls (closed arms), while the remaining two arms are open (open arms). During the test, a mouse is positioned in the central area and allowed to explore the maze for a duration of five min. The duration the mouse spends in the closed arms is compared to the time spent in the open arms to assess levels of anxiety or fear. Mice that are less anxious are more likely to explore the open, exposed arms of the maze, whereas those with higher anxiety levels tend to remain in the closed arms.

#### 3) Golgi staining

Golgi staining was conducted utilizing the FD Rapid Golgi Stain™ Kit, available from FD Neurotechnologies, Inc. (MD, USA). Solutions A and B from the kit were combined in equal parts and left to stand for one week. Mice were deeply anesthetized with isoflurane (Zoetis, UK) and quickly decapitated to extract the entire brain. The brain hemispheres were split and submerged in the A-B solution mix, stored in darkness at room temperature for six days. Subsequently, the brain halves were placed in a cryoprotectant at 4°C, kept in the dark until they sank, with the cryoprotectant being renewed after the first 24 hours. Coronal brain slices, each 200 μm thick, were prepared using a vibratome (Leica Biosystems, Germany) filled with a chilled 6% sucrose solution, cutting from rostral to caudal (bregma −1.34 to −3.80 mm). The sections were immediately affixed to chrome alum-coated slides using solution C and allowed to dry overnight in a dark chamber at room temperature. The staining solution was freshly created by mixing solutions D and E with Milli-Q water in a 1:1:2 ratio. Samples were stained for 10 min and then briefly rinsed with Milli-Q water. Finally, the dried samples were dehydrated through sequential immersions in 70%, 90%, and 100% ethanol (five min each, with 10 min for absolute ethanol) followed by xylene for 15 min, before being cover-slipped.

#### 4) Western blotting

Samples were taken from the cortical and hippocampal tissues of mice and homogenized in a lysis buffer comprising 50 mM Tris-base (pH 7.5), 10 mM sodium pyrophosphate, 1% NP-40, along with protease inhibitors. These samples were then centrifuged at 12,000 rpm for 15 min at 4°C to collect the supernatants. Protein concentrations were measured using the bicinchoninic acid assay from Thermo Scientific. Tissue lysates, each containing 30 μg of protein, were separated by sodium dodecyl sulfate-polyacrylamide gel electrophoresis and transferred onto nitrocellulose membranes (Millipore, Billerica, MA, USA). For immunodetection, the membranes were blocked with 5% skim milk in Tris-buffered saline with 0.1% Tween-20 (TBS-T) at room temperature for one hour, followed by an overnight incubation at 4°C with primary antibodies. The primary antibodies used were: p-Akt (1:1,000, #4058), total Akt (1:1,000, #4691), p-ERK1/2 (1:3,000, #4370), total ERK1/2 (1:3,000, #9102), p-CREB (1:1,000, #9198), total CREB (1:1,000, #9197), p-AMPK (1:1,000, #2537), total AMPK (1:1,000, #2532), p-PSD95 (1:1,000, #45737), and total PSD95 (1:1,000, #3409) from Cell Signaling Technology (Danvers, MA, USA); p-Synapsin1 (S9, 1:1,000, ab76260), p-Synapsin1 (S549, 1:1,000, ab254035), p-Synapsin1 (S603, 1:1,000, ab13879), total Synapsin1 (1:1,000, ab64581), and DCX (1:1,000, ab18723) from Abcam (Cambridge, MA); and β-actin (1:30,000, #A1978) from Sigma-Aldrich. After three 10-min washes with TBS-T at room temperature, the membranes were incubated with secondary antibodies conjugated to horseradish peroxidase, either goat anti-rabbit (Pierce, Rockford, IL, USA) or anti-mouse (Thermo Scientific; 31430). Signals were visualized using the SuperSignal West Pico chemiluminescence kit (Pierce). Bands were imaged with a Molecular Imager ChemiDoc XRS+ (Bio-Rad, Hercules, CA, USA), and band intensities were quantified using Image Lab TM software version 2.0.1 (Bio-Rad, Hercules, CA, USA).

### 4. Exercise program

In this experiment, mice engaged in aerobic treadmill exercises. The intensity levels for the incremental load exercises were adjusted based on a modified version of the animal exercise protocol outlined by Kim et al. [20]. Seven-week-old mice were categorized into four distinct groups: low intensity (n = 10), moderate intensity (n = 10), high intensity (n = 10), and a control group (n = 10). Each group, except the control, participated in treadmill exercises at varying speeds corresponding to their designated intensity levels (Table 1). Specifically, the low-intensity group ran at 4 m/min for 10 min on day one, then at 4 m/min for 10 min followed by 8 m/min for 30 min from the second to the 14th day, and at 4 m/min for 10 min followed by 10 m/min for 25 min from the 15th to the 56th day. The moderate-intensity group began with 8 m/min for 10 min on the first day, progressing to 8 m/min for 10 min and 12 m/min for 25 min from the second to the 14th day, and finally to 8 m/min for 10 min and 15 m/min for 25 min from the 15th to the 56th day. The high-intensity group started at 15 m/min for 10 min on the first day, increased to 15 m/min for 10 min and 20 m/min for 25 min from the second to the 14th day, and then to 15 m/min for 10 min and 23 m/min for 25 min from the 15th to the 56th day. The treadmill sessions were conducted five days a week (Table 1).

### 5. Statistical analysis

Statistical analyses were performed using SPSS version 21.0 (IBM Corporation, Armonk, NY, USA). Results are presented as the mean ± standard error of the mean. The data were analyzed using a one-way analysis of variance, and Dunnett's T3 post-hoc test was subsequently applied. Statistical significance was determined at a *p*-value of less than 0.05.

### 6. Ethical considerations

The protocols for animal use were evaluated and approved by the Institutional Animal Care and Use Committee at Dongguk University Ilsan Hospital (Approval No. IACUC-2019-030) and adhered to the guidelines set by the National Institutes of Health. This study was approved by the Institutional Review Board of Gyeongsang National University (Approval no. 2019030).

## RESULTS

### 1. Moderate-intensity exercise increased locomotor activity

**Figure 1.**
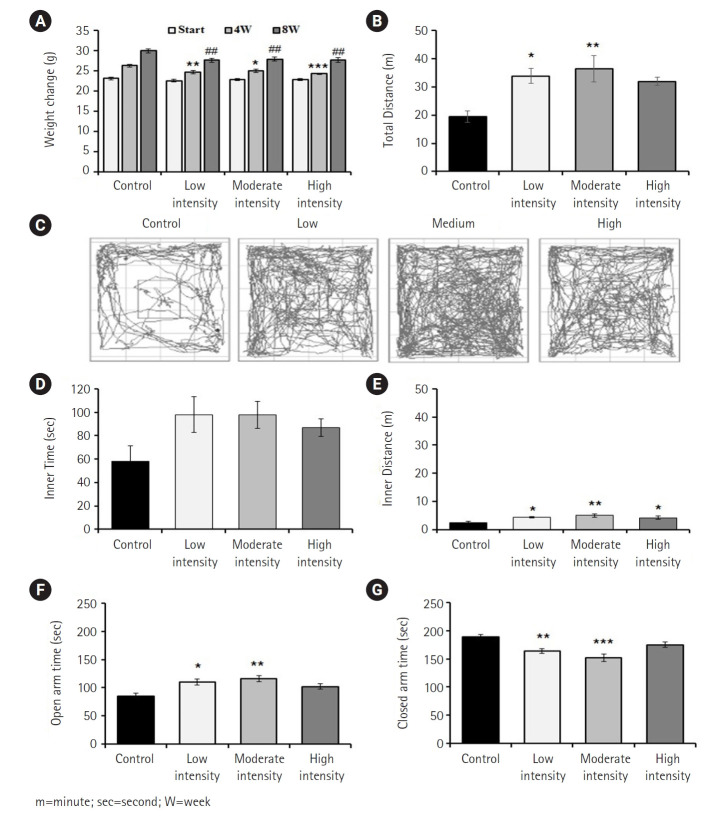
Moderate-intensity exercise increased locomotor activity in young mice. (A) Changes of body total weight gain. The data are expressed as mean ± standard error of the mean (SEM) (n = 8). (***p < .001, **p < .01, *p < .05 vs. Control 4 W; ##*p* < .01 vs. Control 8 W). Anxiety was measured with a behavioral test explanatory diagram, and the distance and time the animal moved in the open field test (B-E) and Elevated plus maze test (F, G). (B) Total distance. (C) Search paths from representative mice in different groups. (D) Inner Time. (E) Inner distance. (F) Open arm time. (G) Closed arm time. The data are expressed as mean ± SEM (n = 8). (***p < .001, **p < .01, *p < .05 vs. Control).

The animals in each group performed treadmill exercise for eight weeks according to the programs listed in Table 1. The mean body weight of the experimental animals before the experiment was 22.89 ± 0.25 g in the high-intensity exercise group, 22.79 ± 0.28 g in the moderate-intensity exercise group, 22.55 ± 0.32 g in the low-intensity exercise group, and 23.13 ± 0.34 g in the control group, and the difference among the groups was not significant. However, a significant change in weight was observed in all exercise groups after four weeks. After eight weeks, The mean body weight of the experimental animals was 27.65 ± 0.54 g in the high-intensity exercise group, 27.85 ± 0.51 g in the moderate-intensity exercise group, 27.60 ± 0.45 g in the low-intensity exercise group, and 29.95 ± 0.47 g in the control group, and all exercise groups gained significantly less weight than the control group (Figure 1-A).

To investigate the effects of treadmill exercise on locomotor activity in the young mice, OFT was performed. The total distance of the low intensity exercise group and the moderate-intensity exercise group was 33.93 ± 2.72 min and 36.48 ± 4.68 min, respectively, which was significantly increased compared to the control group (19.43 ± 1.99). These results indicate that low- and moderate-intensity exercise increased the locomotor activity (Figure 1-B, 1-C).

### 2. Moderate-intensity exercise decreased anxiety

The inner time of the experimental animals was 86.90 ± 7.64 sec in the high-intensity exercise group, 97.9 ± 11.42 sec in the moderate-intensity exercise group, 97.9 ± 15.19 sec in the low-intensity exercise group, and 58.3 ± 12.77 sec in the control group, and The inner time of all exercise group was high, but there was no significant difference compared to the control group. However, the inner distance was 4.27 ± 0.55 min in the high-intensity exercise group, 5.09 ± 0.61 min in the moderate-intensity exercise group, 4.44 ± 0.30 min in the low-intensity exercise group, and 2.46 ± 0.42 min in the control group, and the inner distance of all exercise group was significantly higher than that of the control group. These results suggest that low and moderate-intensity exercise might slightly lower anxiety-like behavior (Figure 1-D, 1-E).

To investigate the effects of treadmill exercise on anxiety in the young mice, an EPM test was additionally performed. The open arm time of the low intensity exercise group and the moderate-intensity exercise group was 110.30 ± 5.32 and 115.70 ± 5.54 sec, respectively, which was significantly increased compared to the control group (85.00 ± 5.31). However, the closed arm time of the low intensity exercise group and the moderate-intensity exercise group was 163.9 ± 4.03 and 152.10 ± 6.50 sec, respectively, which was significantly decreased compared to the control group (188.90 ± 4.37). These results indicate that low and moderate-intensity exercise decreased anxiety in the young mice (Figure 1-F, 1-G).

### 3. Low or moderate-intensity exercise increased neurons

**Figure 2.**
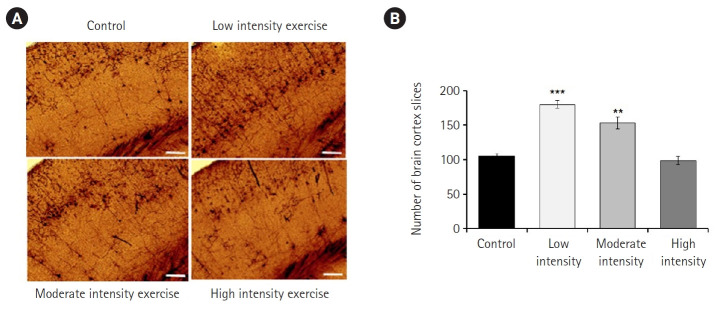
Modified golgi staining showing the differences in brain cortex neurons between control and exercise mice. (A) Image of prefrontal cortex of Golgi–stained brain (scale bar = 100 μm). (B) Summary graph of optical total cell measurement (n = 3). (***p < .001, **p < .01, *p < .05 vs. Control). Image of prefrontal cortex of golgi stained brain (scale bar = 100 μm).

The effects of treadmill exercise on status of pyramidal neurons in the young mice, an Golgi staining was performed. Representative images of Golgi stained neurons for cortex are shown in Figure 2-A. Number of neurons in the low intensity exercise group and the moderate-intensity exercise group was 180.00 ± 10.00 (*p* < .001) and 153.00 ± 14.73 (*p* = .003), respectively, which was significantly increased compared to the control group (105.70 ± 5.13) (Figure 2-B).

### 4. Expression of anxiety-related protein in young mice by exercise

**Figure 3.**
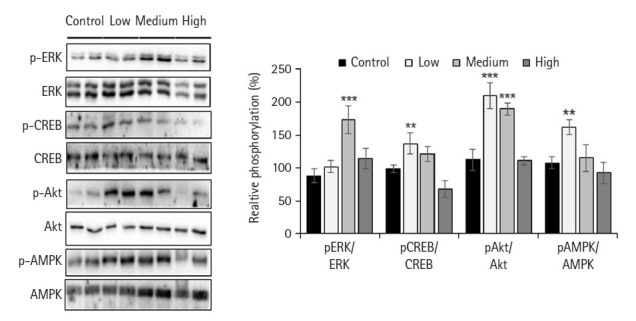
Moderate-intensity exercise increased extracellular signal-regulated kinase (ERK), cyclic adenosine monophosphate response-binding protein (CREB), protein kinase (AKT) and adenosine monophosphate activated protein kinase (AMPK) protein phosphorylation in the hippocampus of young mice. The hippocampal lysates were electrophoresed in 10% sodium dodecyl sulfatepolyacrylamide gel electrophoresis (SDS-PAGE) as well as immunoblotted for each antibody. The concentration of the protein bands was calculated by densitometry. The total bands were normalized to ERK, CREB, AKT, AMPK, and the phosphorylated form was normalized vs. the total form. The data are expressed as mean ± standard error of the mean (SEM) (n = 4).(***p < . 001, **p < .01, *p < .05 vs. Control).

The extracellular signal-regulated kinase (ERK) signaling pathway is involved in anxiety in the hippocampus, lateral amygdala and PFC. Activating the ERK pathway leads to the initiation of transcription factor including cyclic adenosine monophosphate response-binding protein (CREB) and protein kinase (AKT) [21]. Adenosine monophosphate activated protein kinase (AMPK) is also known to bel involved in anxiety and pivotal to sustain neuronal energy level [22]. AMPK is activated by adenosine triphosphate depletion, starvation, hypoxia, and exercise [23]. Immunoblotting was performed in the cortex of young mice to confirm the effect of treadmill exercise on the expression of anxiety-related proteins in young mice. As shown in Figure 3, moderate-intensity exercise group exhibited significant increases in pERK/ERK (F = 25.88, *p* < .001) and pAKT/AKT (F = 53.66, *p* < .001) level compared to the control group. pCREB/CREB (F = 25.27, *p* < .001) and pAMPK/AMPK (F = 16.22, *p* < .001) level were significantly increased in low intensity exercise group compared to control group.

### 5. Neurogenic markers induced in young mice by exercise

**Figure 4.**
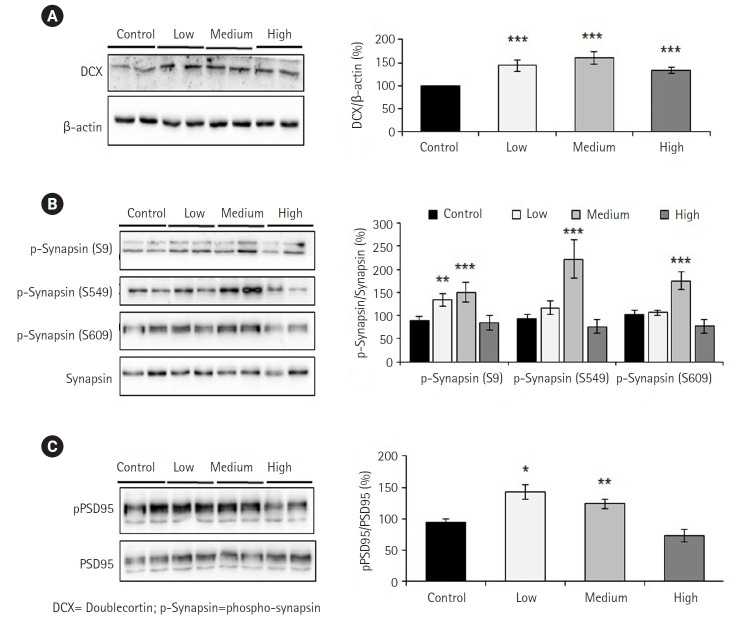
Moderate-intensity exercise increased DCX, p-Synapsin (S9), p-Synapsin (S549), p-Synapsin (S609) and PSD95 protein phosphorylation in the hippocampus of young mice. The hippocampal lysates were electrophoresed in 10% sodium dodecyl sulfate-polyacrylamide gel electrophoresis (SDS-PAGE) as well as immunoblotted for each antibody. The concentration of the protein bands was calculated by densitometry. The total bands were normalized to (A) Doublecortin (Dcx)/β-actin, (B) pSynapsin/Synapsin, (C) pPSD95/PSD95, and the phosphorylated form was normalized vs. the total form. The data are expressed as mean ± standard error of the mean (SEM) (n = 4).(***p < .001, **p < .01, *p < .05 vs. Control).

In our previous experiment, it was observed that the expression of DCX, neurogenic marker, increased in old mice by moderate-intensity exercise [20]. Thus, to investigate the effects of treadmill exercise on DCX expression in the young mice, immunoblotting was performed. As shown in Figure 4, low- and moderate-intensity exercise group exhibited significant up regulation in DCX expression compared to the control group in the hippocampus (F = 29.38, *p* < .001) (Figure 4-A). Moreover, we examined the expression of pSynapsin/Synapsin and pPSD95/PSD95, which is related to neuronal plasticity in brain. Moderate exercise increased the levels of pSynapsin (S9) (F = 18.94, *p* < .001), pSynapsin (S549) (F = 30.62, *p* < .001), and pSynapsin (S609) (F = 38.22, *p* < .001) expression compared to the levels in the cortex of control mice. However, low exercise increased the level of pSynapsin (S9), but not pSynapsin (S549), and pSynapsin (S609) expression (Figure 4-B). Also, low- and moderate-intensity exercise up-regulated the expression level of pPSD95/PSD95 compared to the level of control group in cortex (F = 27.55, *p* < .001) (Figure 4-C).

## DISCUSSION

In this study, the low- and medium-intensity exercise group decreased depression- and anxiety-related behaviors, and increased the neurogenic markers induced in young mice by exercise the anxiety-related ERK, CRER, AKT, and AMPK than the control group and the high-intensity exercise group.

Exercise can help prevent the onset of depression and anxiety in children. For individuals experiencing depression and anxiety, exercise can serve as a method for symptom management [24-27]. Additionally, results from randomized controlled trials indicate that exercise is an effective treatment for depression [28]. Dunn et al. [29] reported that about 40% of people with depression, who were not receiving any other treatments, responded positively to exercise. These findings are similar to more recent results from the REGASSA trial, the most extensive study on exercise and depression, which reported a response rate of approximately 50% [30].

Previous studies [31-33] reported that voluntary wheel running may be effective for enhancing depression-like, anxiety-like, and cognition-like behaviors [31-33]. Adult mice that had wheels running exercise for 3-4 weeks have shown decreases in immobility time in the forced swim test [31,34]. Similarly, investigations focusing on 2 to 4 weeks of running in adult male mice have demonstrated that exercise enhances both the number of center crossings and the duration of time spent in the center of the open field [31,35]. This suggests that long-term exercise reduces anxiety-like behaviors in adult male mice. The benefits of exercise are not only changes in cognitive function, but also decreases depression-like and anxiety-like behaviors.

In many studies, demonstrates that people with depression have decreased levels of brain derived neurotrophic factor (BDNF) [36]. A marker of neuronal growth and plasticity. Decreased nerve regeneration is associated with reduced volume and activity of certain brain regions, including hippocampus, orbital PFC, anterior and posterior singulates, cilia, and temporal lobes, which are observed in patients with depression [37]. Exercise can promote brain plasticity and thus increase hippocampal volume [38]. However, studies that have been reported to change hippocampal volume when depressive patients exercise regularly are very scarce [39]. We found that moderate-Intensity exercise increased locomotor activity and promotes neurogenesis in the cerebral cortex and that DCX, which is associated with neurogenesis, increases in the hippocampus.

Since the 1990s, many studies have been conducted since the beginning of the study to discover the relationship between exercise and neurogenesis. Several previous studies using animals have demonstrated that aerobic exercise increases neurogenesis in the hippocampus [40-42]. While many intervention methods are being studied to promote the secretion of neuroplasticity factors, aerobic exercise of moderate intensity has been shown to have a positive effect on the plasticity of brain neurons. The increase in DCX and BDNF by exercise improves the growth and development of nerve cells in the hippocampus, helping to improve cognitive ability, learning ability, and memory.

In this study, the low- and moderate-intensity exercise groups increased the expression of DCX, a neurogenesis-related factor, and increased the phosphorylation and activity of synapsin and PSD95, which are involved in synaptic plasticity, than the control and high-intensity exercise groups. In this study, high-intensity exercise was not found to be effective. In an experiment with boys aged seven to 15 years with attention-deficit/hyperactivity disorder (ADHD), a single session of high-intensity interval training—consisting of two 10-min sessions with a 1-min break—resulted in significant improvements in CF and sustained attention after 20 min of high-intensity exercise [43]. At moderately high exercise intensities, there was a marked reduction in ADHD symptoms and other comorbidities [44]. The difference between this study and previous studies is that this study involved continuous high-intensity exercise, whereas previous studies used intermittent training that alternated between high-intensity exercise and rest. These results suggest that for children with ADHD, characterized by impulsivity, interval training is a more appropriate form of exercise than continuous high-intensity exercise [45]. This suggests that exercise intensity should be carefully planned, taking into consideration the characteristics of the patient, exercise capacity, and any concomitant diseases or symptoms.

## CONCLUSION

The COVID-19 pandemic is a difficult time, especially for children and adolescents. Interventions for children and adolescents should focus on their psychological and physical health. In this study, the low-intensity and moderate-intensity exercise group reduced depression and anxiety-related behaviors and increased neurogenesis marks in young mice than the control group and the high-intensity exercise group. Based on the results of this study, we propose low and moderate-intensity exercise as an intervention that can support children and adolescents to improve their physical and psychological health and cognition COVID-19 pandemic would be disastrous.

## Figures and Tables

**Table 1.** Exercise Protocol

Intensity	Slope (°)	Duration (day)	Speed (m/min)	Time spent running (min)	Exercise frequency (day/week)
Low	0	1	4	10	5
	0	2-28	4	10	5
			8	30	
	0	29-56	4	10	5
			10	25	
Moderate	0	1	8	10	5
	0	2-28	8	10	5
			12	25	
	0	29-56	8	10	5
			15	25	
High	0	1	15	10	5
	0	2-28	15	10	5
			20	25	
	0	29-56	15	10	5
			23	25	

## Data Availability

The data that support the findings of this study are available from the corresponding author upon reasonable request.
